# Integrative analysis regarding the correlation between GAS2 family genes and human glioma prognosis

**DOI:** 10.1002/cam4.3829

**Published:** 2021-03-12

**Authors:** Chunyan Zhao, Nan Zhang, Xiaoteng Cui, Xinxin Zhang, Yuanyuan Ren, Chao Su, Jinyan He, Wei Zhang, Xiaoming Sun, Jie Yang, Xingjie Gao

**Affiliations:** ^1^ Department of Biochemistry and Molecular Biology Department of Immunology School of Basic Medical Sciences Tianjin Medical University Tianjin China; ^2^ Key Laboratory of Immune Microenvironment and Disease Ministry of Education Key Laboratory of Cellular and Molecular Immunology in Tianjin Excellent Talent Project Tianjin Medical University Tianjin China; ^3^ Laboratory of Neuro‐Oncology Tianjin Neurological Institute Department of Neurosurgery Tianjin Medical University General Hospital and Key Laboratory of Neurotrauma, Variation, and Regeneration Ministry of Education and Tianjin Municipal Government Tianjin China

**Keywords:** GAS2, GAS2L1, GAS2L2, GAS2L3, glioma

## Abstract

**Background:**

Emerging oncogenes were reportedly linked to the complicated subtypes and pathogenesis of clinical gliomas. Herein, we first comprehensively explored the potential correlation between growth‐arrest‐specific two family genes (*GAS2*, *GAS2L1*, *GAS2L2*, *GAS2L3*) and gliomas by bioinformatics analysis and cellular experiments.

**Methods:**

Based on the available datasets of TCGA (The Cancer Genome Atlas), CGGA (Chinese Glioma Genome Atlas), and Oncomine databases, we performed a series of analyses, such as gene expression, survival prognosis, DNA methylation, immune infiltration, and partner enrichment. We also utilized two glioma cell lines to conduct the colony formation and wound‐healing assay.

**Results:**

*GAS2L3* gene was highly expressed in glioma tissues compared to normal brain tissues (*p* < 0.05). We further observed the relationship between the high expressed GAS2L3 and poor clinical prognosis of brain low‐grade glioma (LGG) cases in our Cox proportional hazard model (hazard ratio [HR] = 0.1715, *p* < 0.001). Moreover, DNA hypomethylation status of *GAS2L3* was correlated with the high expression of *GAS2L3* in LGG tissues and the poor clinical prognosis of primary glioma cases (*p* < 0.05). We also found that the high expression of *GAS2L3* was associated with the infiltration level of immune cells, especially the T cells (*p* < 0.0001). Functional enrichment analysis of *GAS2L3*‐correlated genes and interaction partners further indicated that *GAS2L3* might take part in the occurrence of glioma by influencing a series of biological behaviors, such as cell division, cytoskeleton binding, and cell adhesion. Additionally, our cellular experiment data suggested that a highly expressed *GAS2L3* gene contributes to the enhanced proliferation and migration of glioma cells.

**Conclusion:**

This study first analyzed the potential role of GAS2 family genes, especially *GAS2L3*, in the clinical prognosis and possible functional mechanisms of glioma, which gives a novel insight into the relationship between *GAS2L3* and LGG.

## Graphical abstract

Compared with *GAS2*, *GAS2L1*, and *GAS2L2*, there is a stronger correlation between *GAS2L3* gene expression and glioma prognosis. DNA hypomethylation of *GAS2L3* may contribute to the high expression level of *GAS2L3* and the poor clinical prognosis of glioma. *GAS2L3* gene is capable of influencing the proliferation and migration ability of glioma cells and may be linked to a series of immune cell infiltration or cell division‐associated events in the etiology and biology of glioma.

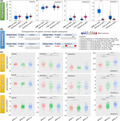

## 1 INTRODUCTION

The interaction between congenital high‐risk factors and environmental carcinogens contributes to the occurrence of clinical gliomas, the most common malignant primary brain tumor.^1^, ^2^ Although there are many grading systems for gliomas, the most commonly used is the grading system developed by the World Health Organization (WHO), namely, WHO I, II, III, and IV.^2^, ^3^ Brain low‐grade glioma (LGG) is the glioma of WHO II–III grade, whereas glioblastoma multiforme (GBM) is the most malignant form of glioma with the WHO IV grade and poor prognosis.^3^, ^4^, ^5^ The aim of this study is thus to comprehensively analyze the potential functional links of the growth‐arrest‐specific 2 (GAS2) family genes with the pathogenesis or clinical prognosis of gliomas.

Members of GAS2 family include GAS2, GAS2‐like 1 (GAS2L1), GAS2‐like 2 (GAS2L2), and GAS2‐like 3 (GAS2L3).^6^, ^7^ These members have been reported to be implicated in the cellular polarization, motility, or centrosome dynamics, through affecting the cytoskeleton system.^6^, ^7^, ^8^ Very recently, we have published a review article regarding the structures and functions of GAS2 family.^9^ Even though GAS2L3 was essential for the morphogenesis and development of brains,^10^ there was still no evidence regarding the potential association between GAS2 family members and clinical brain gliomas.

TCGA database includes the multiple‐genomics data of gliomas (https://portal.gdc.cancer.gov/).^11^, ^12^ The CGGA database contains the available brain tumor datasets, such as the whole‐exome sequencing, DNA methylation, mRNA sequencing, and matched clinical data (http://www.cgga.org.cn/). Herein, we first investigated the expression patterns of GAS2 family genes, including *GAS2*, *GAS2L1*, *GAS2L2*, and *GAS2L3*, in the glioma tissues, and explored the potential correlation between the expression level of GAS2 family genes and the clinical prognosis of glioma cases within TCGA or CGGA databases. Also, we considered a series of factors (e.g., DNA methylation, genetic mutation, immune infiltration, etc.) to investigate the potential molecular mechanisms regarding the effect of GAS2 family genes on the pathogenesis of gliomas. Moreover, we performed cellular experiments to study the relationship between the expression of the *GAS2L3* gene and the in vitro proliferation and migration ability of glioma cells.

## 2 MATERIALS AND METHODS

### 2.1 Gene mapping and protein structure analysis

We analyzed the genome location of GAS2 family genes, including *GAS2*, *GAS2L1*, *GAS2L2*, and *GAS2L3*, using the “Genome Data viewer” function (https://www.ncbi.nlm.nih.gov/genome/gdv/) of the National Center for Biotechnology Information (NCBI). Then, the “HomoloGene” function (https://www.ncbi.nlm.nih.gov/homologene/) of NCBI was utilized for a conserved functional domain analysis of these four GAS2 family members among the different species. In addition, we used the basic local alignment search tool (BLAST, https://blast.ncbi.nlm.nih.gov/Blast.cgi) of NCBI to perform the protein sequence alignment analysis of human GAS2 (NP_808221.1), GAS2L1 (NP_006469.2), GAS2L2 (NP_644814.1), and GAS2L3 (NP_777602.1).

### 2.2 Gene expression analysis

Next, we investigated the expression pattern of the GAS2 family genes in the different tumors or specific tumor subtypes through the Tumor IMmune Estimation Resource (TIMER) tool (https://cistrome.shinyapps.io/timer/), as reported previously.^13^ Due to the very limited data of adjacent nontumor tissues in The Cancer Genome Atlas‐brain lower‐grade glioma/glioblastoma multiforme (TCGA‐LGG/GBM) project (http://tcga‐data.nci.nih.gov/tcga/), we included the normal brain tissues (*n* = 207) of The Genotype‐Tissue Expression (GTEx) databases as normal controls using a standard processing pipeline Gene Expression Profiling Interactive Analysis, version 2 (GEPIA2) webserver (http://gepia2.cancer‐pku.cn/#analysis).^14^, ^15^ The median expression of tumor and normal samples was also displayed in the bodymap. We also logged into the online UALCAN portal (http://ualcan.path.uab.edu/index.html)^16^ and obtained the expression difference of GAS2 family genes in the LGG or GBM cases from different ethnic populations. Besides, we pooled a total of eight datasets in the Oncomine database (https://www.oncomine.org/resource/login.html) to analyze the expression difference of GAS2 family genes between normal control and glioblastoma. Further, the association between GAS2 family genes expression and glioma WHO classifications (WHO II, III, and IV) was analyzed using the three datasets (array_301, seq_325, seq_693) of the Chinese Glioma Genome Atlas (CGGA) database.

### 2.3 Survival prognosis analysis

Based on the datasets of the TCGA‐LGG/GBM project, we conducted the overall survival (OS) and disease‐free survival (DFS) analysis to analyze the potential correlation between GAS2 family gene expression and the prognosis of glioma patients through the GEPIA2 tool.^13^ The results were visualized by the Kaplan–Meier curve and survival map. The factor of the race was also included for the prognosis analysis through the UALCAN portal. Furthermore, we employed the TIMER tool to perform a multivariate cox regression analysis, and the covariables of age, gender, race, and tumor purity were used in the Cox proportional hazard model. Additionally, we carried out a series of survival analyses based on the datasets of the primary or recurrent glioma cases within the CGGA database.

### 2.4 DNA methylation analysis

Based on the Methyl_159 dataset of CGGA, we analyzed the potential association between DNA methylation status of GAS2 family genes and the glioma WHO classification or the clinical prognosis of primary/recurrent glioma. Meanwhile, we applied the tools of MEXPRESS (https://mexpress.be/) and MethSurv (https://biit.cs.ut.ee/methsurv/) to investigate the correlation between gene expression/clinical prognosis and the methylation status of different sites, based on the datasets of TCGA‐LGG/GBM project. The results were visualized by the Kaplan–Meir plot and the heat map of clustering analysis of individual cytosine–phosphate–guanine (CpG) islands, respectively.

### 2.5 Mutation analysis

We utilized the cBioPortal tool (https://www.cbioportal.org/)^17^, ^18^ to analyze the mutation features of the GAS2 family members for the glioma cases of TCGA‐LGG/GBM project. The results of alteration frequency, mutation type, and CNV information were shown. We also performed the OS and disease/progression‐free survival (D/PFS) analyses to explore the potential correlation between the mutation status and the prognosis of overall cancer patients through cBioPortal.^13^ Additionally, we investigated the mutation profile of *GAS2*, *GAS2L1*, *GAS2L2*, and *GAS2L3* for the glioma cases of the CGGA WEseq_286, and the result was visualized as an oncoprint.

### 2.6 Immune infiltration analysis

Based on the datasets of TCGA‐LGG/GBM, we explored the potential relationship between the expression of GAS2 family genes and the infiltration level of immune cells, including B cell, CD8^+^ T cell, CD4^+^ T cell, macrophage, neutrophil, and dendritic cell, through the TIMER tool. Furthermore, we utilized the GEPIA2 tool to analyze the potential correlation between GAS2 family gene expression and the immune infiltration status of resident memory T cell, effector memory T cell, effector T cell, effector Treg T cell, exhausted T cell, and Th1‐like T cell, respectively. We applied the non log scale for calculation and the log‐scale axis for visualization. Spearman's correlation test was performed to calculate the correlation coefficient.

### 2.7 GAS2L3‐correlated gene enrichment analysis

We utilized three approaches, including GEPIA2, UALCAN, and LinkedOmics (http://www.linkedomics.org/admin.php),^19^ to obtain the top 200 *GAS2L3*‐correlated genes on basis of the dataset of TCGA‐LGG. Then, we performed an intersection analysis to identify the common genes by the online Venn tool (http://bioinformatics.psb.ugent.be/webtools/Venn/). Next, the expression correlation between *GAS2L3* and the common genes was detected by the “Correlation Analysis” function of GEPIA2 and CGGA, based on the datasets of the TCGA‐LGG and CGGA array_301/seq_325/seq_693. As reported previously,^13^ we utilized the R software (R‐3.6.1 version) to perform the gene ontology (GO) enrichment analysis through the “clusterProfiler” package (http://www.bioconductor.org/packages/release/bioc/html/clusterProfiler.html). Also, we performed the Kyoto Encyclopedia of Genes and Genomes (KEGG) pathway analysis through the approach of the database for annotation, visualization, and integrated discovery (DAVID) (https://david.ncifcrf.gov/) and ggplot2 R package (https://cran.r‐project.org/web/packages/ggplot2/index.html).

### 2.8 GAS2L3 interaction partner analysis

We utilized the biological general repository for interaction datasets (BioGRID) (https://thebiogrid.org/)^20^ to further analyze the potential interacting proteins of GAS2L3. The relevant interaction network was displayed by the layout of the concentric circle. Also, we performed a GO enrichment analysis of these GAS2L3 binding proteins to identify the possible biological functions. The data was visualized by the emapplot function of “clusterProfiler” R package (http://www.bioconductor.org/packages/release/bioc/html/clusterProfiler.html).

### 2.9 Cell and plasmid

Two human glioma cell lines (N9, N33), kindly provided by Prof. Chunsheng Kang, were cultured in the Dulbecco's Modified Eagle Medium: Nutrient Mixture F‐12 (DMEM: F12, 01‐172‐1ACS, Biological Industries) with 10% fetal bovine serum (FBS, 04‐001‐1A, Biological Industries). Human embryonic kidney 293T (HEK 293T) cell line was purchased from the American Type Culture Collection (ATCC) and cultured according to the instructions of the manufacturer.

The plasmid of the pLVX‐IRES‐Puro vector (632183, Clontech) was generously gifted by Prof. Lei Shi. The coding sequence of *GAS2L3* cDNA (NM_174942.3) was cloned into the pLVX‐IRES‐Puro vector with a Flag sequence at the 3ʹ end with XhoI and XbaI sites to obtain a pLVX‐IRES‐Puro‐GAS2L3‐Flag plasmid. Two shRNAs targeting the *GAS2L3* gene were cloned into a TRC2‐pLKO‐Puro vector (shGAS2L3‐#1 and shGAS2L3‐#2). The shRNA sequences: (shGAS2L3‐#1) 5′‐CCGGAGTCCGTTCTAAATTGCCAAACTCGAGTTTGGCAATTTAGAACGGACTTTTTTG‐3′ (Forward), 5′‐AATTCAAAAAAGTCCGTTCTAAATTGCCAAACTCGAGTTTGGCAATTTAGAACGGACT‐3′ (Reverse); (shGAS2L3‐#2) 5′‐CCGGCGTGCCAGTTAGTATTCCAAACTCGAGTTTGGAATACTAACTGGCACGTTTTTG‐3′ (Forward), 5′‐AATTCAAAAACGTGCCAGTTAGTATTCCAAACTCGAGTTTGGAATACTAACTGGCACG‐3′ (Reverse).

The lentivirus particles were produced from the cell culture supernatant after the cotransfection of the above lentiviral expression plasmid and two envelope expressing plasmids in HEK 293Tcells. The N9 or N33 cells were infected with lentivirus for 48 h and treated with 2 µg/ml of puromycin (Solarbio) for 1 week. Finally, positive stable cell lines were obtained.

### 2.10 Western blotting assay

A western blotting assay was performed as previously described.^21^ The anti‐GAPDH (Proteintech Group), anti‐Flag (Sigma‐Aldrich), and anti‐GAS2L3 (Abnova) antibodies were utilized.

### 2.11 Colony formation assay

We conducted the colony formation assay to explore the potential role of *GAS2L3* expression in the proliferation of glioma stable cell lines, including the N9‐IRES‐Vector, N9‐IRES‐GAS2L3‐Flag, N33‐pLKO‐Vector, N33‐pLKO‐shGAS2L3‐#1, and N33‐pLKO‐shGAS2L3‐#2 cells. Briefly, the glioma cells were seeded at a concentration of 1 × 10^3^ cells per well in 6‐well plates and cultured for 2 weeks. Then, cells were fixed with 4% paraformaldehyde (Sigma‐Aldrich) and stained with 0.2% crystal violet (Solarbio). Megascopic cell colonies were counted by Image J 2X software (Bethesda, MD, USA).

### 2.12 Wound‐healing assay

A wound‐healing assay was performed to explore whether the knockdown of the *GAS2L3* gene affected the migration of glioma cells. Briefly, N33‐pLKO‐Vector, N33‐pLKO‐shGAS2L3‐#1, and N33‐pLKO‐shGAS2L3‐#2 cells were seeded at 90% confluent in 6‐well plates, respectively. Three vertical wounds were scratched per well. The relative migration status was detected using an inverted microscope at the designated time (0, 24, 48, and 72 h). According to previously described,^22^, ^23^ the cell‐free wound area of 0 h after the scratch was set to 1, and the value of the migration rate was calculated by measuring the reduction percentage of wound area by Image J 2X software (Bethesda, MD, USA), namely, wound closure (%).

### 2.13 Statistical analysis

We performed the Student's *t* test and an ANOVA test, using the SPSS 13.0 software (IBM) or GraphPad Prism 8.0.2 (San Diego, California USA). Differences with a two‐tailed *p* value lower than 0.05 were considered statistically significant.

## 3 RESULT

### 3.1 Genetic mapping and protein structure analysis data

In the present study, we focused on the human GAS2 family genes, including the *GAS2* (chromosome 11), *GAS2L1* (chromosome 22), *GAS2L2* (chromosome 17), and *GAS2L3* (chromosome 12) (Figure S1A). The protein structures of GAS2, GAS2L1, GAS2L2, and GAS2L3 are conservative among the different species (e.g., *Homo sapiens*, *Pan troglodytes*, *Macaca mulatta*, etc.) and commonly include the domains of calponin homology (CH, cl00030) and growth‐arrest‐specific protein 2 (GAS2, cl02524) (Figure S1B). We further performed a protein sequence alignment analysis and found that the N‐terminus structures of GAS2 (313aa), GAS2L1 (681aa), GAS2L2 (880aa), and GAS2L3 (694aa) in *H. sapiens* exhibit the similarities (Figure S1C).

### 3.2 Gene expression data

We analyzed the expression pattern of GAS2 family genes in different tumor tissues and adjacent controls in the TCGA database. As shown in Figure S2, there exists the distinct expression status of *GAS2*, *GAS2L1*, *GAS2L2*, and *GAS2L3* genes for the different tumors. For instance, compared with normal tissues of TCGA‐BRCA (Breast invasive carcinoma), we observed a lower expression level of *GAS2* and *GAS2L2* genes (Figure S2, *p* < 0.001), but a high expression level of GAS2L3 (*p* < 0.001) in the tumor tissues. Additionally, the *GAS2L3* gene was highly expressed in other tumors, such as cholangio carcinoma (CHOL), esophageal carcinoma (ESCA), kidney renal clear cell carcinoma (KIRC), stomach adenocarcinoma (STAD), and uterine corpus endometrial carcinoma (UCEC), compared with adjacent controls (Figure S2, *p* < 0.001).

**FIGURE 1:**
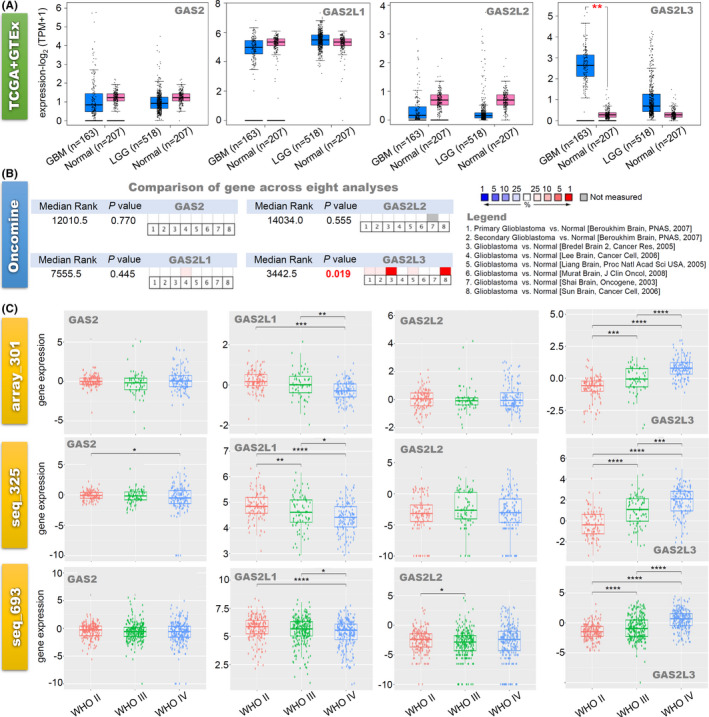
The expression analysis of GAS2 family genes in glioma. (A) We analyzed the expression levels of *GAS2*, *GAS2L1*, *GAS2L2*, and *GAS2L3* in glioma tissues in the TCGA‐LGG/GBM project and normal brain tissues in the GTEx database. (B) Based on the eight analyses of the Oncomine database, we comprehensively analyze the expression difference of GAS2 family genes between normal control and glioblastoma. (C) We investigated the association between GAS2 family gene expression and glioma World Health Organization (WHO) classifications (WHO II, III, and IV), based on the three datasets (array_301, seq_325, seq_693) of the CGGA. *p < 0.05; **p < 0.01; ***p < 0.001; ****p < 0.0001

**FIGURE 2:**
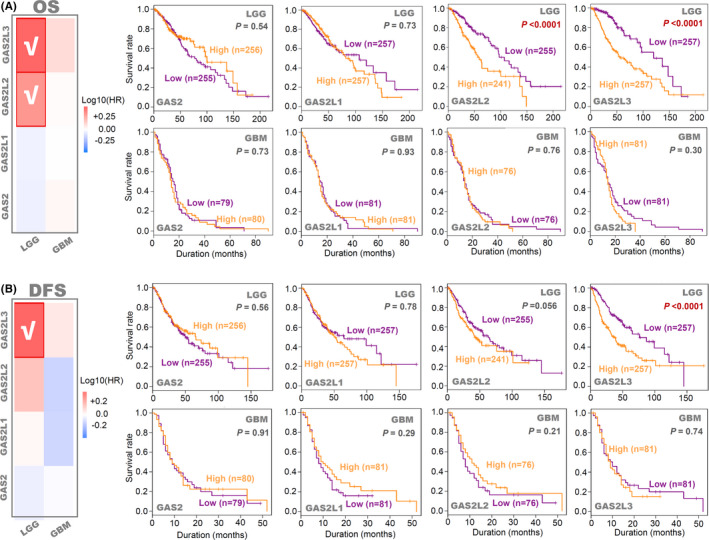
Correlation between GAS2 family genes expression and glioma prognosis (TCGA‐LGG/GBM). Based on the data of the TCGA‐LGG/GBM project, we performed the (A) OS and (B) DFS analysis to study the correlation of GAS2 family genes expression with the prognosis of glioma through the GEPIA2 tool. The survival map and Kaplan–Meier curve were provided, respectively

Even though there is a trend of high expression in the LGG group (*n* = 518), compared with the GTEx controls (*n* = 207), we did not observe the statistical difference (Figure 1A). Also, we did not observe the expression difference of GAS2L3 for LGG cases among the different ethnic populations (Figure S3A). Nevertheless, GAS2L3 is highly expressed in the Caucasian (Figure S5A, *n* = 139, *p* < 1.0e‐12) and African‐American (*n* = 10, *p* < 1.7e‐03) cases of GBM, compared with adjacent normal tissues (*n* = 5). When compared with the normal controls of GTEx database (*n* = 207), *GAS2L3* gene also showed a higher expression level in the TCGA‐GBM group (*n* = 163) (Figure 2A, *p* < 0.01). Moreover, our pooled analysis of eight datasets of Oncomine showed a higher expression level of the *GAS2L3* gene in glioblastoma tissues than the normal tissues (Figure 1B, *p* = 0.019). Based on three CGGA datasets (array_301, seq_325, seq_693), we further analyzed the expression differences of GAS2 family genes in different WHO classifications (WHO II, III, and IV). As shown in Figure 1C, from WHO II to WHO IV, there was an increased expression trend of the *GAS2L3* gene. The above data hints that the *GAS2L3* gene expression might be associated with the occurrence and development of gliomas. Apart from *GAS2L3*, we did not observe the strong evidence supporting the significant expression difference of the *GAS2*, *GAS2L1*, and *GAS2L2* genes between glioma cases and negative controls (Figure 1A,B, Figures S3A and S4A).

### 3.3 Survival analysis data

Based on the datasets of TCGA‐LGG/GBM, we analyzed the potential correlation between GAS2 family genes expression and clinical prognosis of glioma cases. As shown in Figure 2A,B, the highly expressed *GAS2L2* gene was linked to the poor OS prognosis of LGG (*p* < 0.0001), while the high expression of the *GAS2L3* gene was associated with the poor OS and DFS prognosis of LGG (*p* < 0.0001). When the race factor was included, we observed the positive conclusion for the association between the expression of *GAS2L2* (Figure S3B, *p* < 0.0001) or *GAS2L3* (*p* < 0.0001) and the prognosis of LGG cases, but not GBM cases (Figure S4B). Furthermore, we included the covariables of age, gender, race, and tumor purity for a Cox proportional hazard model and observed the association between the poor prognosis of LGG and high expression of *GAS2L2* (Table S1, Cox_*p* < 0.001, HR = 1.315), *GAS2L3* (Cox_*p* < 0.001, HR = 1.526), and low‐expression *GAS2L1* (Cox_*p* = 0.010, HR = 0.642).

Besides, we performed a prognostic analysis on the three datasets (array_301, seq_325, seq_693) of CGGA and observed a correlation between the high expression of *GAS2L3* and poor clinical prognosis of primary glioma cases (Figure S5A–C, all *p* < 0.0001). In contrast, the low expression of *GAS2L1* was associated with a poor prognosis of primary glioma cases (Figure S5A, *p* < 0.0001; Figure S5B, *p* < 0.0001). These results suggested that increased expression of the *GAS2L3* gene may predict an unfavorable prognosis of low‐grade or primary glioma patients.

### 3.4 DNA methylation analysis data

**FIGURE 3:**
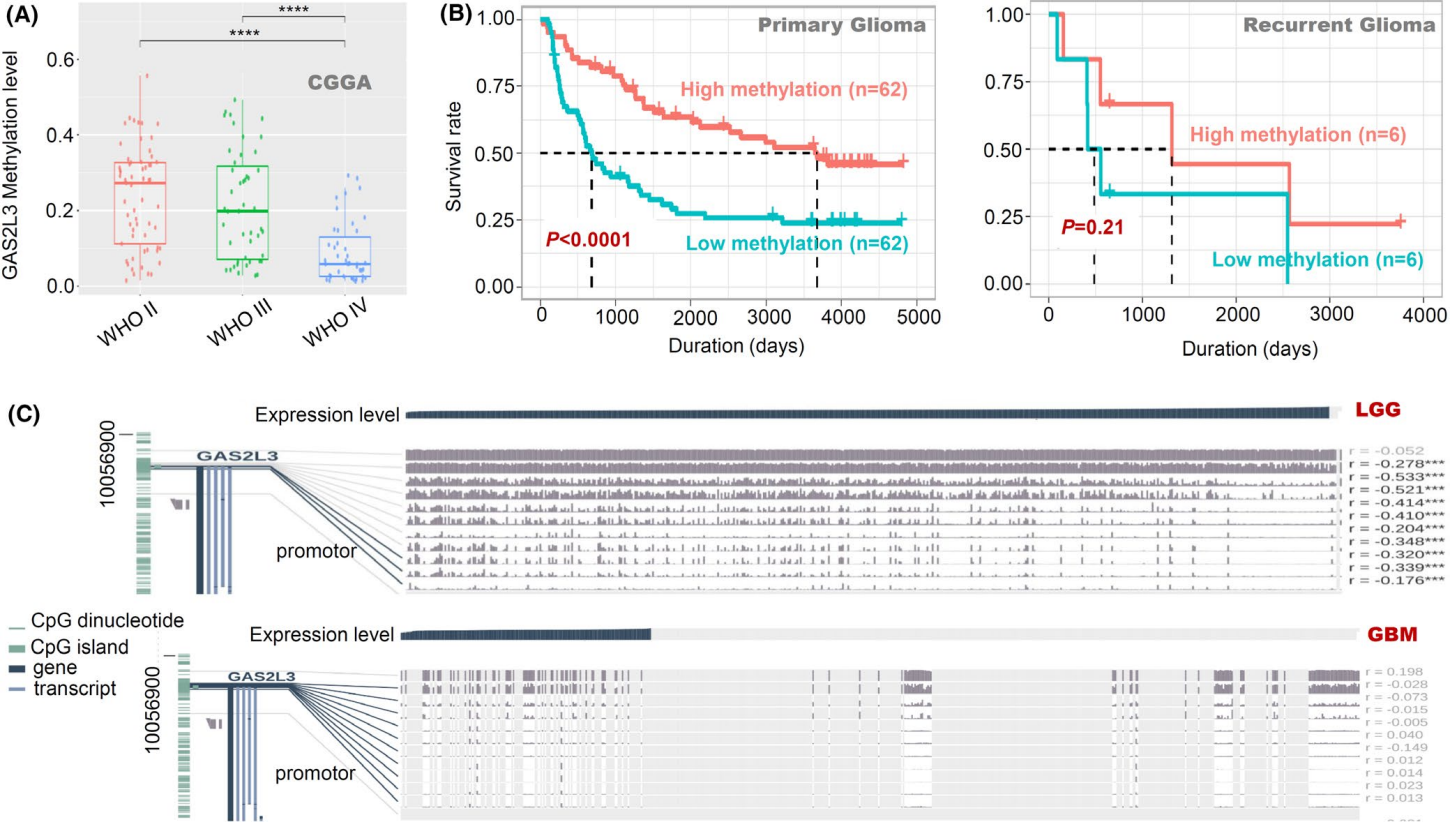
DNA methylation analysis of the *GAS2L3* gene in glioma patients. Based on the Methyl_159 dataset of CGGA, we investigated (A) the potential association of DNA methylation status of *GAS2L3* with the glioma WHO classifications or (B) the clinical prognosis of primary/recurrent glioma. (C) We also utilized the tools of MEXPRESS to analyze the correlation between gene expression and the methylation status of different sites, based on the data of the TCGA‐LGG/GBM project, respectively. ***p < 0.001; ****p < 0.0001

Next, the methylation status of GAS2 family genes in the glioma cases was analyzed. Even though we failed to observe an obvious DNA methylation status of *GAS2*, *GAS2L1*, and *GAS2L2* (data not shown) in the CGGA Methyl_159 dataset, a reduced DNA methylation level of *GAS2L3* was negatively correlated with the glioma WHO classification (Figure 3A) and led to a poor prognosis of primary glioma (Figure 3B, *p* < 0.0001). Besides, we observed the correlation between the DNA hypomethylation of multiple sites and the high expression level of *GAS2L3* in the TCGA‐LGG group (Figure 3C, *p* < 0.001), but not TCGA‐GBM (Figure 3C). Moreover, *GAS2L3* DNA hypomethylation at specific sites (such as cg13069247, cg16653538, cg12203636, etc.) was associated with poor clinical outcomes for LGG (Figure S6), but not GBM (Figure S7).

### 3.5 Mutation analysis data

We also analyzed the mutation status of *GAS2*, *GAS2L1*, *GAS2L2*, and *GAS2L3* in the glioma samples of TCGA‐LGG/GBM or CGGA‐WEseq_286 project, respectively. As shown in Figure S8A, the alteration frequency of these four members in the TCGA‐LGG/GBM project was lower than 1.2%, and no significant mutation of *GAS2L3* was detected in the TGCG‐GBM project, while ~0.2% amplification mutations of *GAS2L3* were presented in the TGCA‐GBM project. Figure S8B presents the specific mutation type and copy number variations information. Similarly, the mutation frequency for all members in the CGGA‐WEseq_286 project was less than 1% (Figure S9). Additionally, we did not observe the statistical correlation between the mutation status and clinical prognosis of all tumor cases in the TCGA project (Figure S8C,D, all *p* > 0.05).

### 3.6 Immune infiltration analysis data

**FIGURE 4:**
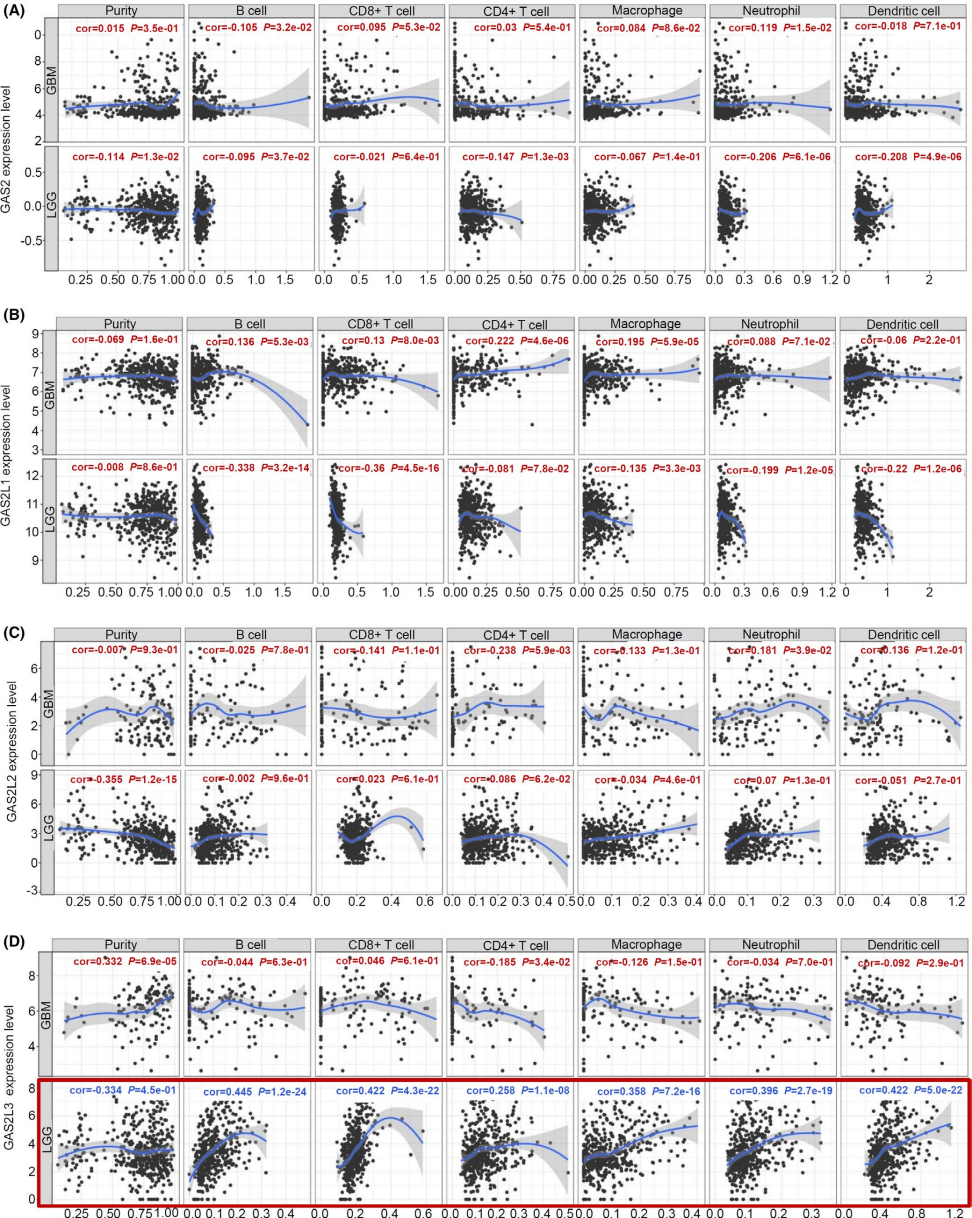
Correlation analysis between GAS2 family genes expression and immune cell infiltration level in glioma patients (TIMER). Based on the data of TCGA‐LGG/GBM, we utilized the TIMER tool to analyze the potential correlation between the expression level of (A) *GAS2*, (B) *GAS2L1*, (C) *GAS2L2*, and (D) *GAS2L3*, and infiltration level of the immune cells, including B cell, CD8^+^ T cell, CD4^+^ T cell, macrophage, neutrophil, and dendritic cell, respectively

Due to the links of infiltrating immune cells in the tumor microenvironment with glioma cells,^24^, ^25^ we explored the potential correlation between the GAS2 family genes expression and the infiltration level of different immune cells by TIMER. As shown in Figure 4, the expression level of *GAS2L3* gene in the cases of TCGA‐LGG was positively correlated with the infiltration level of B cell (cor = 0.445), CD8^+^ T cell (cor = 0.422), CD4^+^ T cell (cor = 0.258), macrophage (cor = 0.358), neutrophil (cor = 0.396), and dendritic cell (cor = 0.422) (all *p* < 0.0001). Furthermore, we detected the positive correlation between *GAS2L3* gene expression and the infiltration level of resident memory T cell (*R* = 0.5), effector memory T cell (*R* = 0.36), effector T cell (*R* = 0.37), effector Treg T cell (*R* = 0.25), exhausted T cell (*R* = 0.3), and Th1‐like T cell (*R* = 0.35), in the TCGA‐LGG (all *p* < 0.0001), but not TCGA‐GBM project (Figure S10). These suggested the potential functional links of *GAS2L3* expression and the immune cell infiltration for the brain lower grade glioma tissues.

### 3.7 Enrichment analysis of *GAS2L3*‐associated partners

**FIGURE 5:**
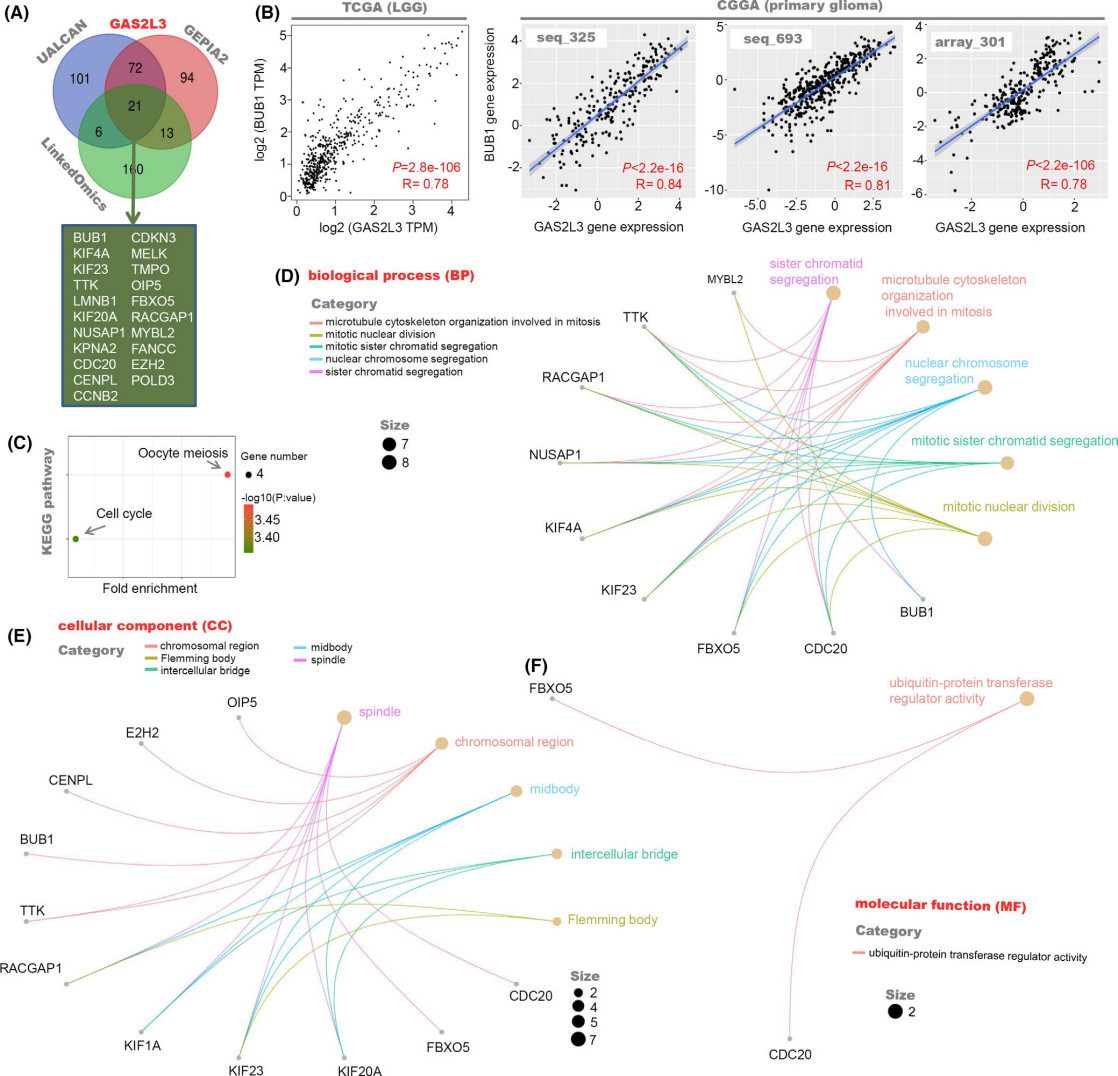
*GAS2L3*‐correlated gene enrichment analysis in LGG patients. (A) We screened out three lists of the top 200 *GAS2L3*‐correlated genes, through UALCAN, GEPIA2, and LinkedOmics, and performed the intersection analysis by the Venn tool. (B) We utilized the “Correlation Analysis” function of GEPIA2 or CGGA to analyze the expression correlation between *GAS2L3* and *BUB1* gene. (C) KEGG pathway analysis was then performed by the DAVID tool and ggplot2 package. The (D) BP, (E) CC, (F) and MF data in GO analysis were also provided

To further explore the potential molecular mechanism regarding the *GAS2L3* overexpression in the pathogenesis of LGG, we identified and enriched the *GAS2L3*‐correlated targeting genes. As shown in Figure 5A, we screened out three lists of the top 200 *GAS2L3*‐correlated genes, through three ways of UALCAN, GEPIA2, and LinkedOmics, and obtained a total of 21 common genes (e.g., *BUB1*, *KIF4F*, *CDKN3*, etc.) through an intersection analysis. Then, we conducted a series of correlation analyses on the glioma cases within the TCGA and CGGA databases and observed a strong correlation between *GAS2L3* and these common genes. Figure 5B presents a high positive relationship between the *GAS2L3* and *BUB1* gene as an example (all *R* > 0.75, *p* < 0.0001). KEGG pathway analysis indicated that most of these genes are related to the cell cycle and oocyte meiosis pathways (Figure 5C). GO analysis data (Figure 5D–F) also supported the functional link of *GAS2L3* expression with a series of cell division‐associated cellular components (e.g., spindle, midbody, etc.) and biological processes (e.g., sister chromatid segregation, mitotic nuclear division, etc.).

Besides, as shown in Figure S11A, we utilized the BioGRID analysis to obtain a total of 20 potential interacting proteins of GAS2L3 with the experimental evidence of “affinity capture‐mass spectrometric,” “two‐hybrid,” “reconstituted complex”, or “proximity Label‐mass spectrometric.” And the MF (molecular function) data of our GO enrichment analysis (Figure S11B) suggested that these proteins mainly were associated with the biological processes of cell adhesion, actin‐binding, and protein phosphatase activity.

### 3.8 The role of GAS2L3 expression in the proliferation and migration of glioma cells

**FIGURE 6:**
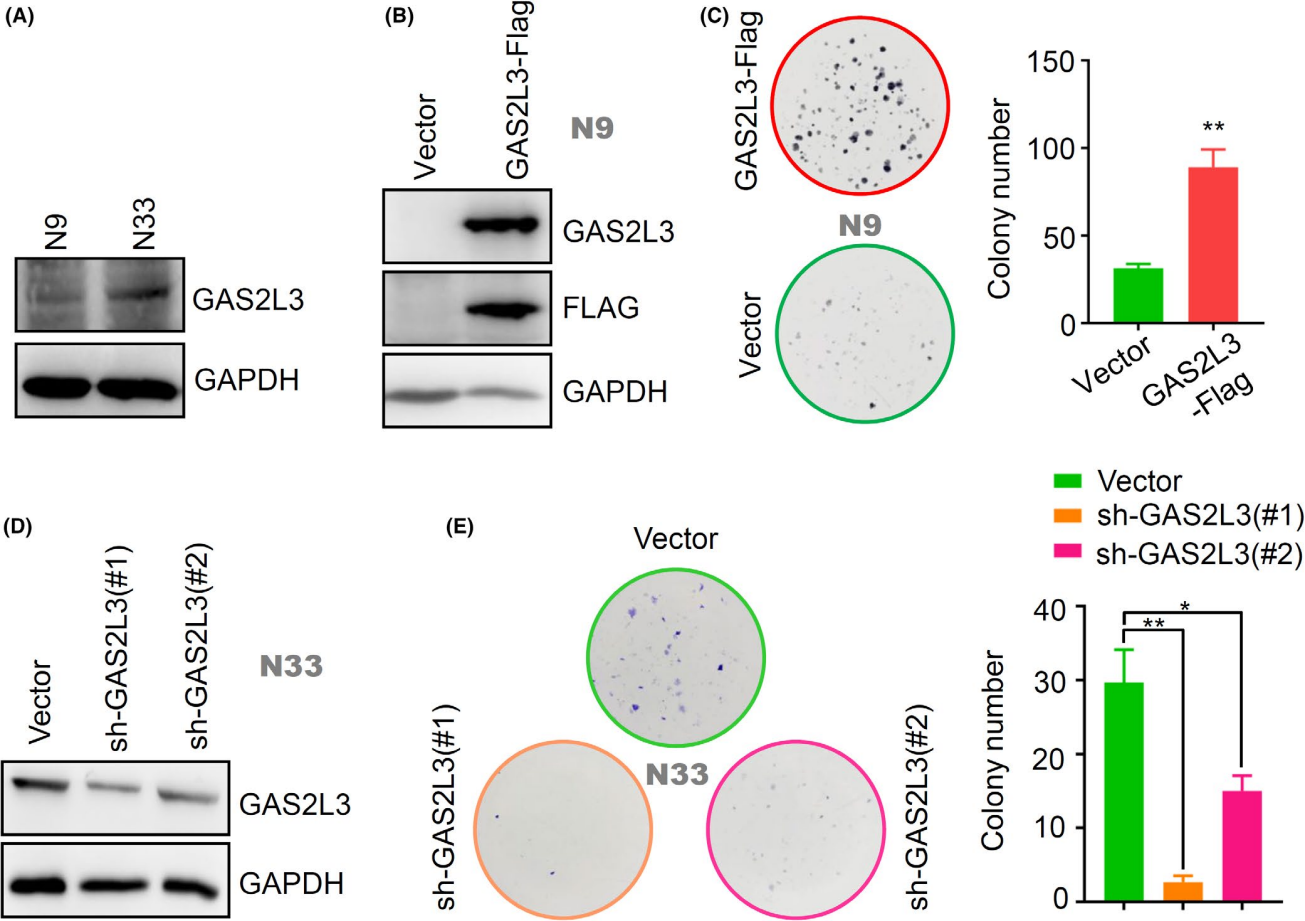
The effect of *GAS2L3* gene expression on the proliferation of glioma cells. (A) A western blotting assay was performed to detect the expression level of GAS2L3 protein in N9/N33 glioma cell lines, using anti‐GAS2L3 or anti‐GAPDH antibody as the control. (B) The expression status of the GAS2L3‐Flag fusion protein was detected in N9‐IRES‐Vector and N9‐IRES‐GAS2L3‐Flag stable cell lines through a western blotting assay by anti‐GAS2L3, anti‐Flag, or anti‐GAPDH antibody. (C) Then, a colony formation assay was conducted. Similarly, we also performed a (D) western blotting assay and (E) colony formation assay using the N33‐pLKO‐Vector, N33‐pLKO‐shGAS2L3‐#1, and N33‐pLKO‐shGAS2L3‐#2 cells. The colony number was calculated and analyzed by Student's *t* test (***p < 0.001) or ANOVA test (*p < 0.05, **p < 0.01)

Finally, we analyzed the association between *GAS2L3* gene expression and the proliferation or migration processes of two human glioma cell lines (N9, N33). Western blotting data suggested that the expression level of the GAS2L3 protein in N33 cells is slightly higher than that in N9 cells (Figure 6A). Then, we performed a colony formation assay using the N9‐IRES‐Vector and N9‐IRES‐GAS2L3‐Flag stable cell lines. As shown in Figure 6B, GAS2L3‐Flag fusion protein was expressed in the GAS2L3 cells but not N9‐IRES‐Vector cells, and a higher colony number was observed in the GAS2L3‐Flag overexpression group, compared with the vector group (Figure 6C, *p* < 0.001). Furthermore, we observed a reduced expression level of the *GAS2L3* gene (Figure 6D) and decreased colony number (Figure 6E, *p* < 0.05) in the N33‐pLKO‐shGAS2L3‐#1 and N33‐pLKO‐shGAS2L3‐#2 cells, compared with the N33‐pLKO‐Vector. These data supported the links between *GAS2L3* expression and the cellular proliferation of glioma cells.

**FIGURE 7:**
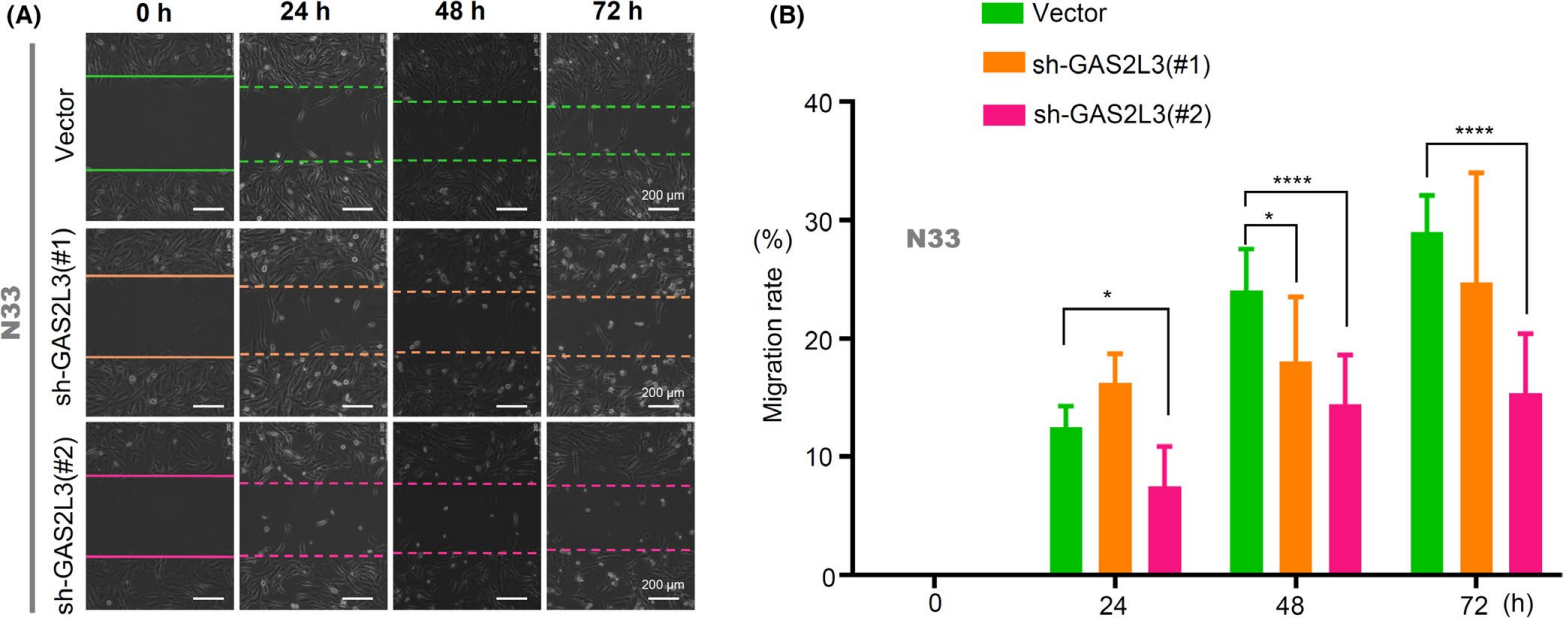
The effect of *GAS2L3* gene expression on the migration of glioma cells. N33‐pLKO‐Vector, N33‐pLKO‐shGAS2L3‐#1, and N33‐pLKO‐shGAS2L3‐#2 cells were used for the wound‐healing assay. (A) The images were provided. Scale bar, 200 µm. (B) The migration rate were calculated and analyzed by ANOVA test (*p < 0.05, ****p < 0.0001)

Additionally, we downregulated the expression of the *GAS2L3* gene in N33 cells for the wound‐healing assay. The results (Figure 7A) showed that, compared with the vector group, the down‐regulation of the *GAS2L3* gene in the sh‐GAS2L3‐#1 and sh‐GAS2L3‐#2 groups led to a reduced migration trend of glioma cells, especially at the point of 48 h (Figure 7B, *p* < 0.05). Therefore, *GAS2L3* expression is associated with the migration ability of glioma cells.

## 4 DISCUSSION

*GAS2* gene was named because it was specifically expressed at growth arrest of mammalian NIH3T3 cells.^26^, ^27^ Also, other GAS2 family members, including GAS2L1, GAS2L2, and GAS2L3, were reported to be linked to the cell cycle or division events. For instance, the knockdown of the *GAS2L1* gene could lead to the presence of aberrant cell division and nuclear abnormalities.^28^ Abnormal expression of the *GAS2L3* gene influences the process of cell abscission, the final stage of cell division.^29^ Nevertheless, there was still very limited evidence regarding the potential association between GAS2 family genes and certain clinical diseases, especially tumors. In the present study, we are interested in analyzing the possible biological behavior of GAS2 family genes in the occurrence and development of glioma by bioinformatics analysis of the available public databases and functional cell assays.

Regarding the *GAS2L1* gene, we observed a negative correlation between the gene expression and the WHO grading or the poorer clinical OS prognosis (array_301, seq_325) of glioma cases within the CGGA database. Nevertheless, after integrating the eight datasets within the Oncomine database, we failed to detect a statistical difference of *GAS2L1* expression between normal controls and glioblastoma tissues. Based on the data of the TCGA‐LGG/GBM project, there was no correlation between *GAS2L1* expression and the clinical prognosis of gliomas cases. Additionally, we only observed the potential association between the *GAS2* gene expression and the prognosis of glioma cases in the seq_325 of the CGGA database. Similarly, the high expression of *GAS2L2* was only associated with the clinical OS prognosis of glioma patients in the TCGA‐LGG project. Therefore, our findings did not provide strong evidence regarding the correlation between the expression of *GAS2*, *GAS2L1*, and *GAS2L2* and the clinical prognosis of glioma cases. Even though this, we still cannot rule out the potential relationship between the three members and other types of tumor, due to the fact of statistical expression difference between other tumor tissues and adjacent control tissues.

Compared with other GAS2 family members, there was a strong correlation between the *GAS2L3* gene expression and the prognosis of glioma patients. After analyzing the datasets of Oncomine, TCGA, and GTEx databases, we observed a higher expression level of *GAS2L3* in the glioma tissues than the normal controls. In addition, *GAS2L3* expression was positively correlated with WHO gradings of glioma cases within the CGGA database. Interestingly, TCGA‐based survival curve analysis suggested that high expression of *GAS2L3* is associated with the clinical prognosis of LGG, but not GBM. CGGA‐based data analysis indicated that high expression of *GAS2L3* is associated with poor clinical prognosis of the primary glioma cases, but not the recurrent glioma cases. Hence, we focused on the correlation between *GAS2L3* and gliomas, especially LGG.

The mutation of epigenetic regulator genes is essential for the subclassification or treatment of glioma.^1^, ^2^ A pathogenic variant of the *GAS2L2* gene was reportedly associated with a genetic defect in ciliary orientation and mucociliary clearance.^30^ The gene mutation status of GAS2 family genes in glioma was thus analyzed. Nevertheless, we observed the very low mutation frequency of four GAS2 family members for the glioma cases in either the TCGA or CGGA database. Due to the limited data, we only analyzed the correlation between GAS2 family member mutation and the clinical prognosis of overall cancer patients and obtained negative results. Therefore, genetic mutations may not be involved in the function mechanism of GAS2 family members.

Besides gene mutation, DNA methylation was considered. DNA methylation is associated with the gene expression, clinical prognosis, or the pathological classification of gliomas^1^, ^31^; with regard to *GAS2L3*, we detected the correlation between DNA hypomethylation and the high expression level, or the poor prognosis of LGG cases. Additionally, we found that the infiltration of immune cells (such as B cell, effector T cell, effector Treg T cell, or exhausted T cell) is correlated with the expression level of *GAS2L3*. Nevertheless, we did not observe the positive results in the dataset of GBM. Hence, DNA methylation and immune infiltration are more likely to contribute to the molecular mechanism of *GAS2L3* involved in the pathogenesis of LGG, but not glioblastoma multiforme.

Several studies reported the role of *GAS2L3* in the cell division event.^9^, ^29^, ^32^, ^33^, ^34^ For instance, the data of *GAS2L3*‐deficient mice indicates an important role of *GAS2L3* in the cardiomyocyte cytokinesis during heart development.^34^ Similarly, as a target gene of the dimerization partner, RB‐like, E2F, and multi‐vulval class B (DREAM) complex with maximal expression in G2/mitosis, *GAS2L3* is essential for the completion of cytokinesis in mammalian cells.^32^ In line with the above cell cycle/division‐associated functional attributes of *GAS2L3*, our cellular experiment data supported the association between the high *GAS2L3* expression and an increased proliferation and migration capabilities within glioma cells. Also, the 21 *GAS2L3*‐correlated genes in our enrichment analysis (Figure 5A) were mainly involved in fundamental processes of the cell cycle and proliferation.^35^ Besides, these genes were overlapped with the target genes of the DREAM complex,^36^ and chromosomal instability (CIN) 25 signature.^37^ These suggested that the high expression o*f GAS2L3* is not specific to one cancer type. As the data in Figure 2, there is an elevated *GAS2L3* expression for the tumors of CHOL, ESCA, KIRC, STAD, and UCEC.

The treatment of highly cytotoxic alpha‐emitter‐immunoconjugates can result in the downregulation of *GAS2L3* expression in gastric cancer HSC45‐M2 cells.^38^ It is meaningful to investigate the potential effect of *GAS2L3* expression on the medication treatment and clinical treatment of LGG, and other factors, such as TP53 mutation, 1p19q codeletion, and isocitrate dehydrogenase (IDH) 1/2 mutation, should be fully considered as well.

In summary, compared with *GAS2*, *GAS2L1*, and *GAS2L2*, there is a stronger correlation between *GAS2L3* gene expression and glioma prognosis. DNA hypomethylation of *GAS2L3* may contribute to the high expression level of *GAS2L3* and the poor clinical prognosis of glioma cases. Moreover, the *GAS2L3* gene can affect the proliferation and migration ability of glioma cells and may be associated with a series of immune cell infiltration or cell division‐associated events in the etiology and biology of glioma.

## CONFLICT OF INTEREST

The authors declare no competing financial interests.

## Supporting information

Fig S1

Fig S2

Fig S3

Fig S4

Fig S5

Fig S6

Fig S7

Fig S8

Fig S9

Fig S10

Fig S11

Table S1

## Data Availability

The data that support the findings of this study are available on request from the corresponding author.
