# Supplementary figures and images for: Integrative analysis regarding the correlation between GAS2 family genes and human glioma prognosis
Source: Cancer Med. 2021 Mar 12;10(8):2826–39. doi: 10.1002/cam4.3829 (PMC8026934; doi:10.1002/cam4.3829)

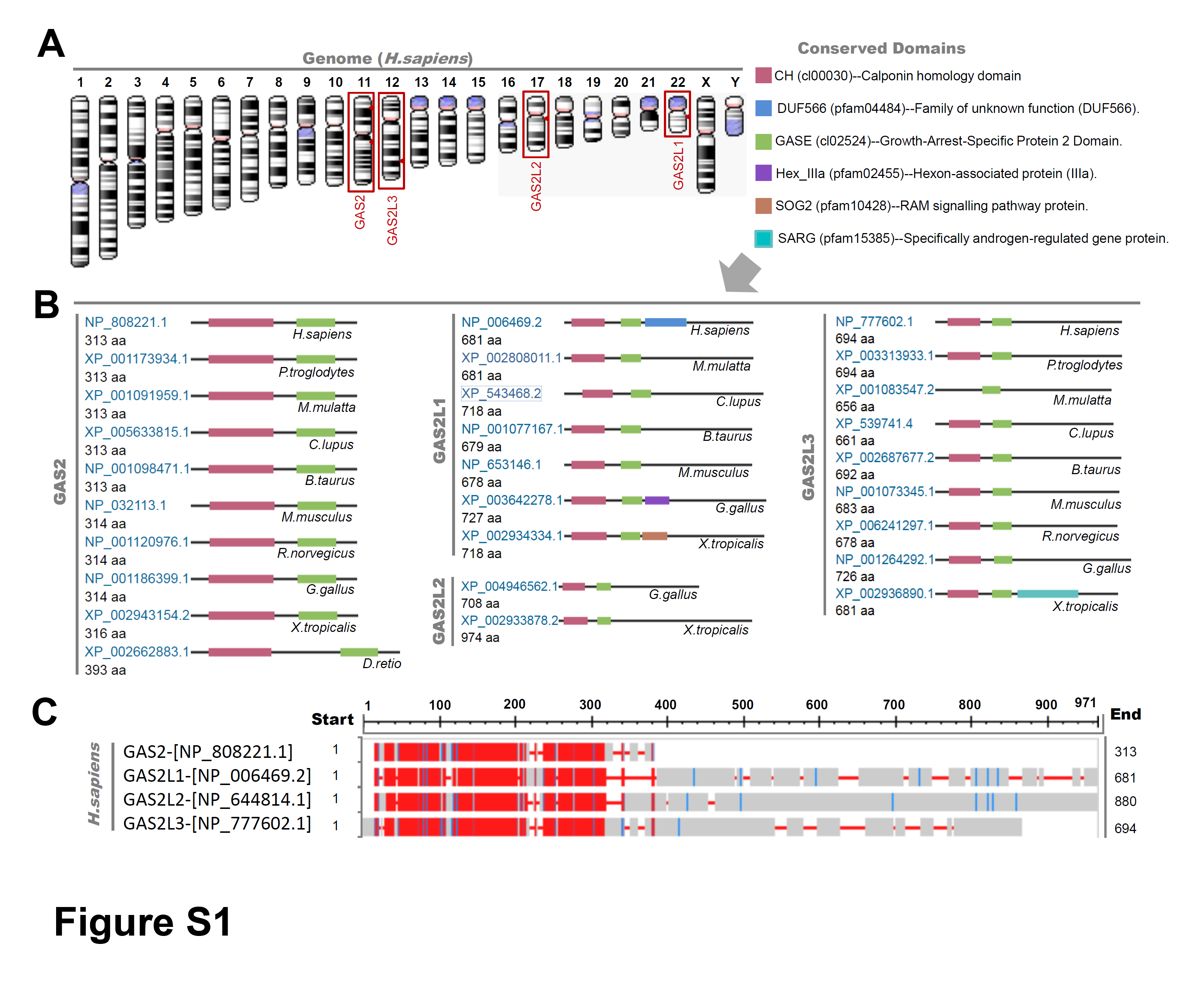

Supplement: Supplementary file 1 — Fig S1 [file CAM4-10-2826-s009.tif]

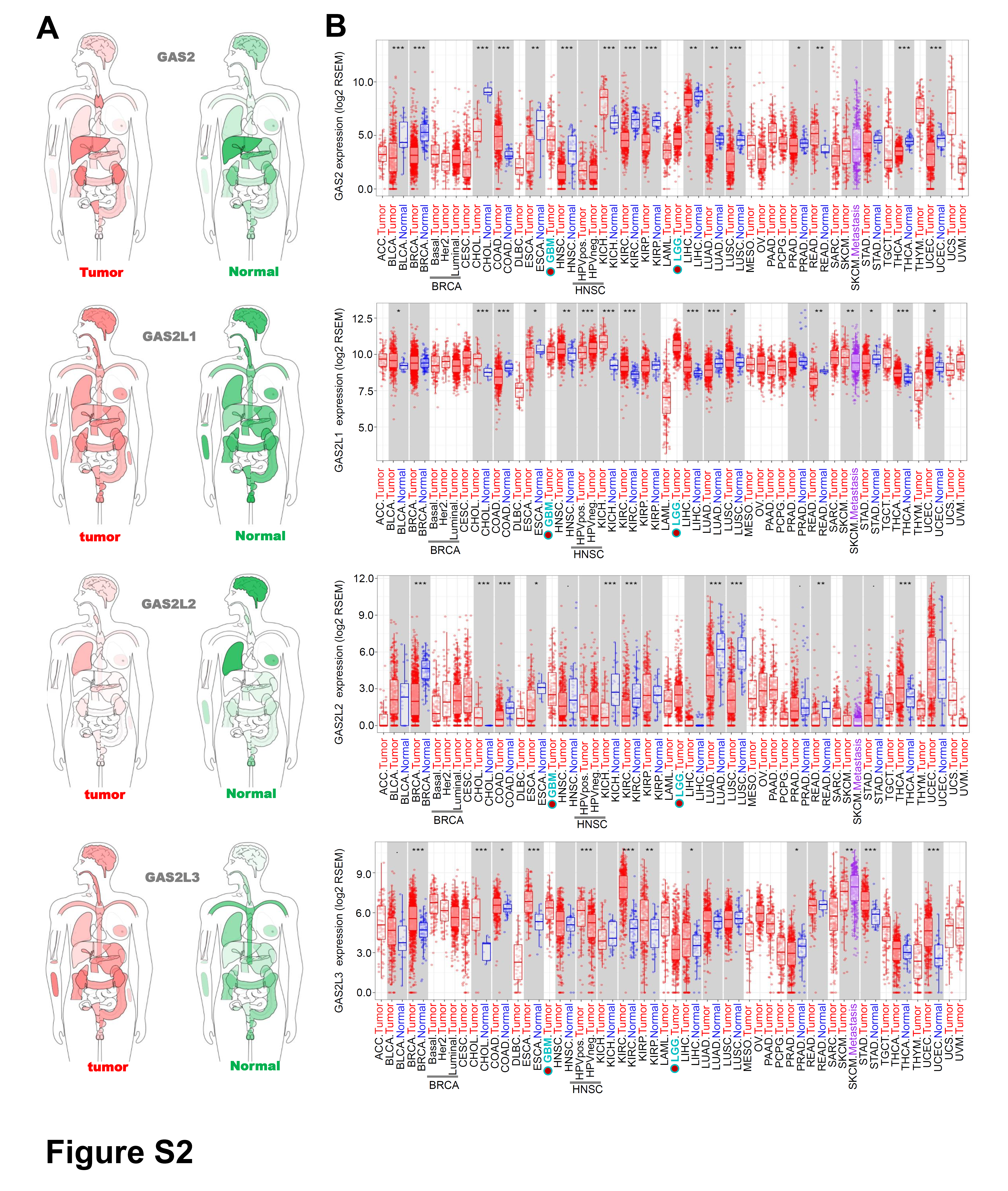

Supplement: Supplementary file 2 — Fig S2 [file CAM4-10-2826-s007.tif]

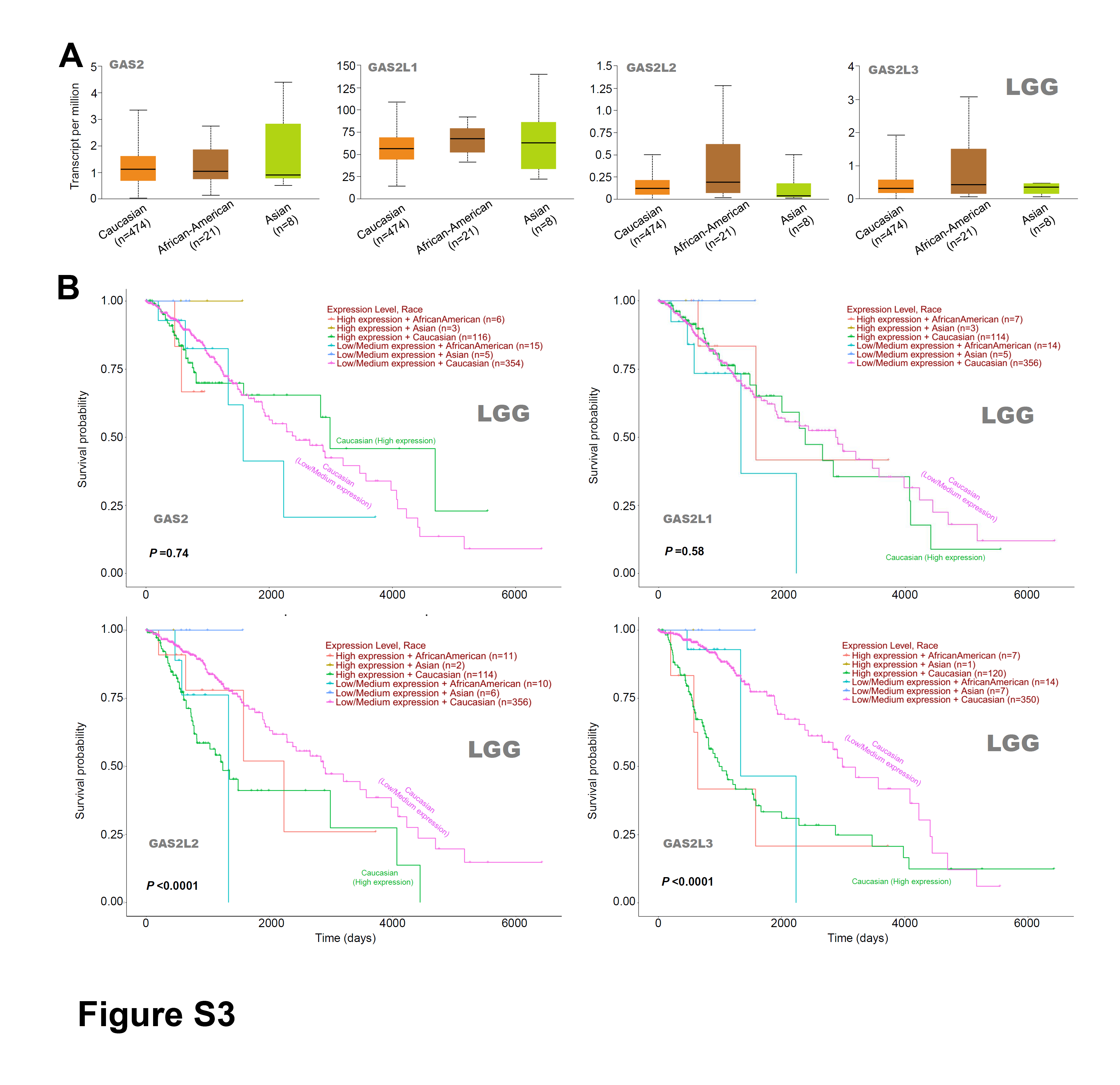

Supplement: Supplementary file 3 — Fig S3 [file CAM4-10-2826-s012.tif]

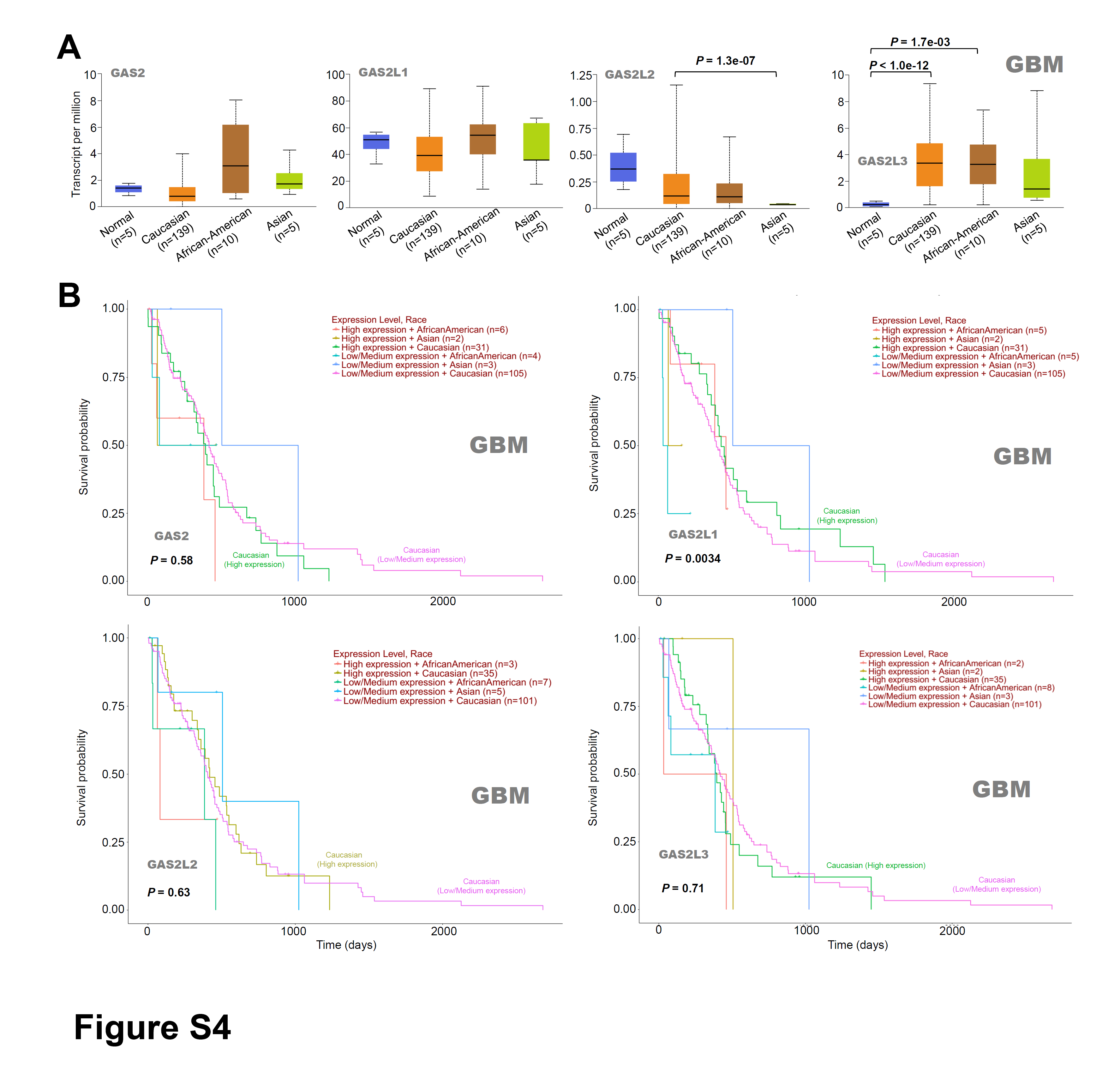

Supplement: Supplementary file 4 — Fig S4 [file CAM4-10-2826-s006.tif]

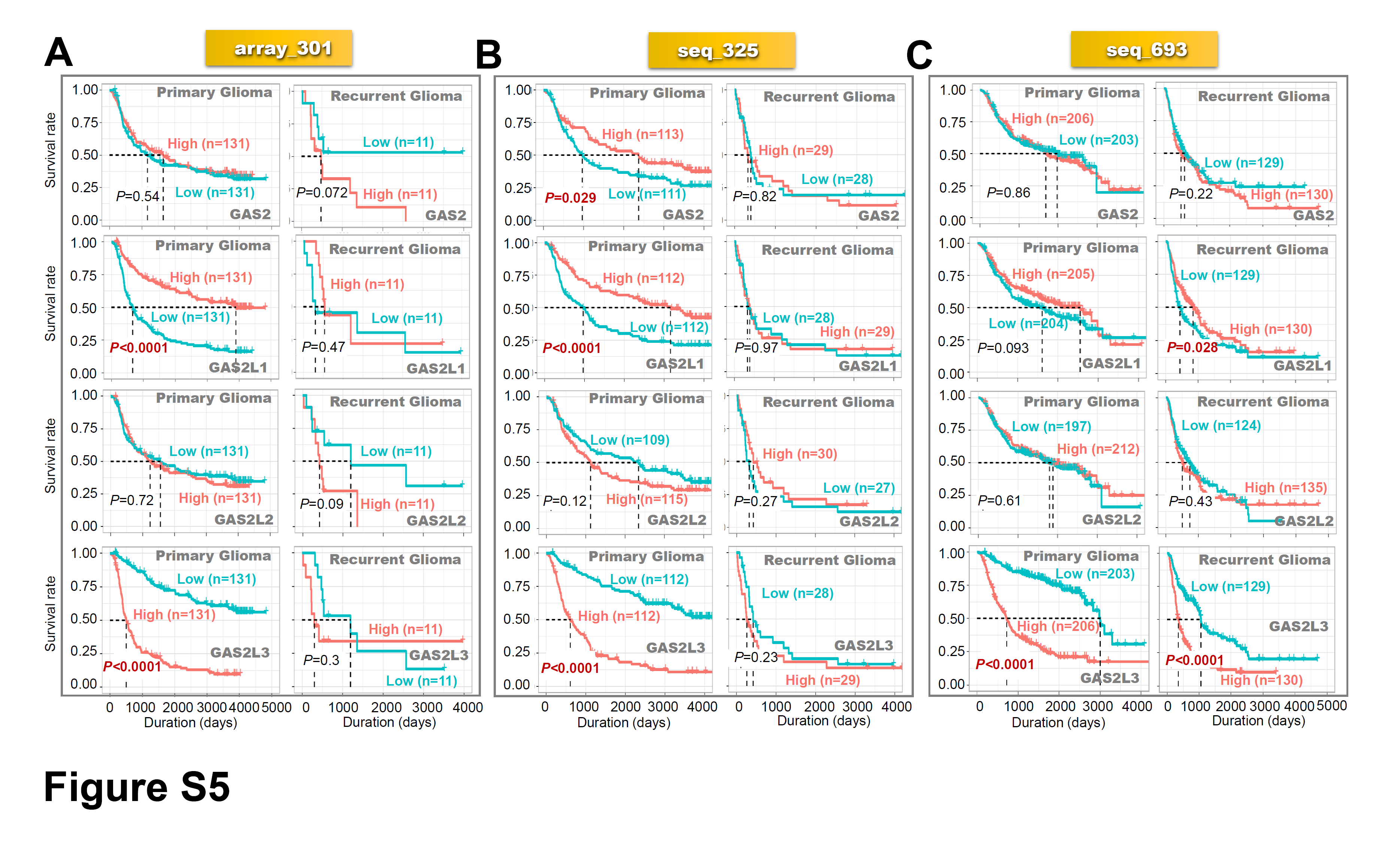

Supplement: Supplementary file 5 — Fig S5 [file CAM4-10-2826-s001.tif]

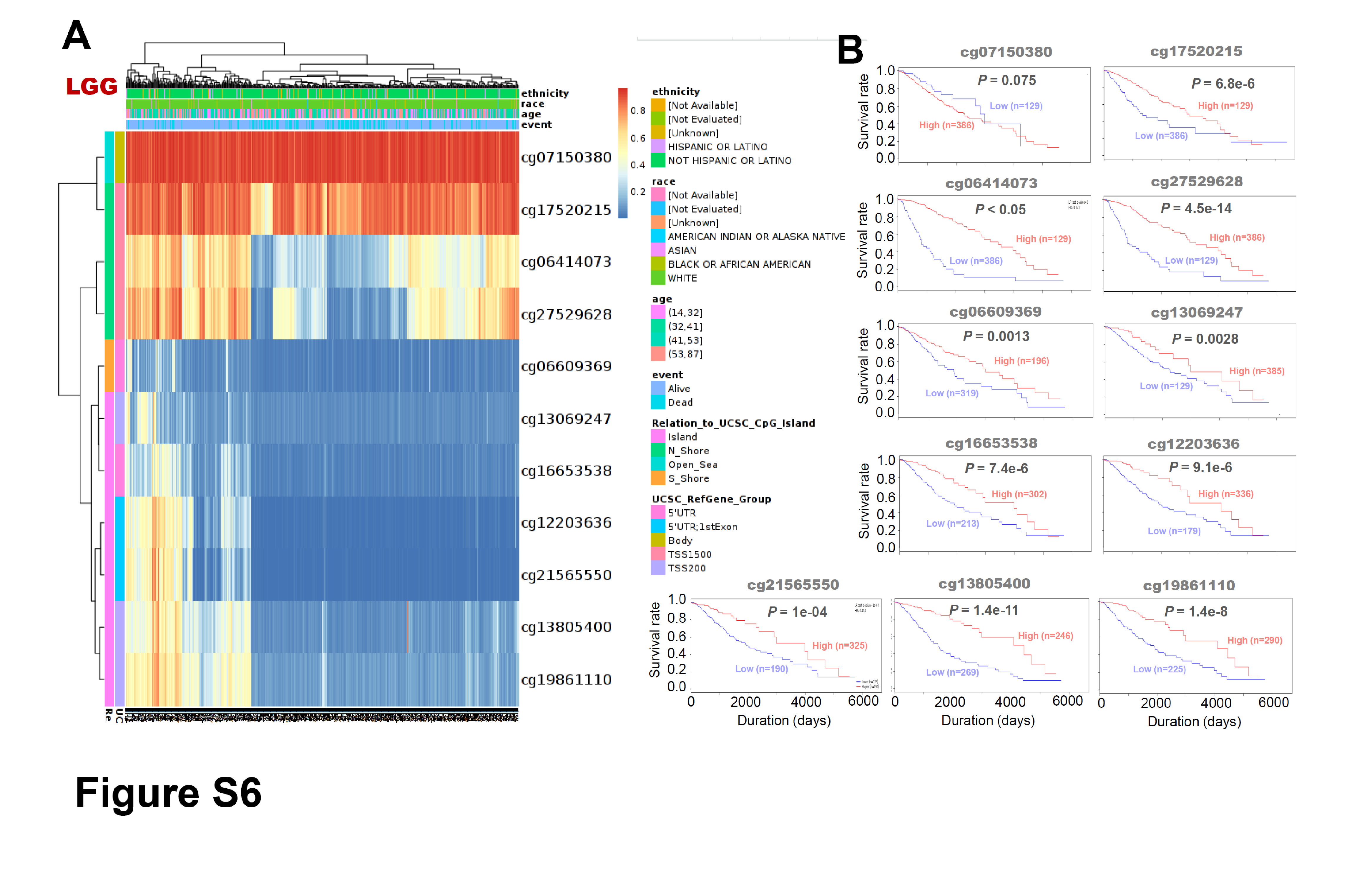

Supplement: Supplementary file 6 — Fig S6 [file CAM4-10-2826-s005.tif]

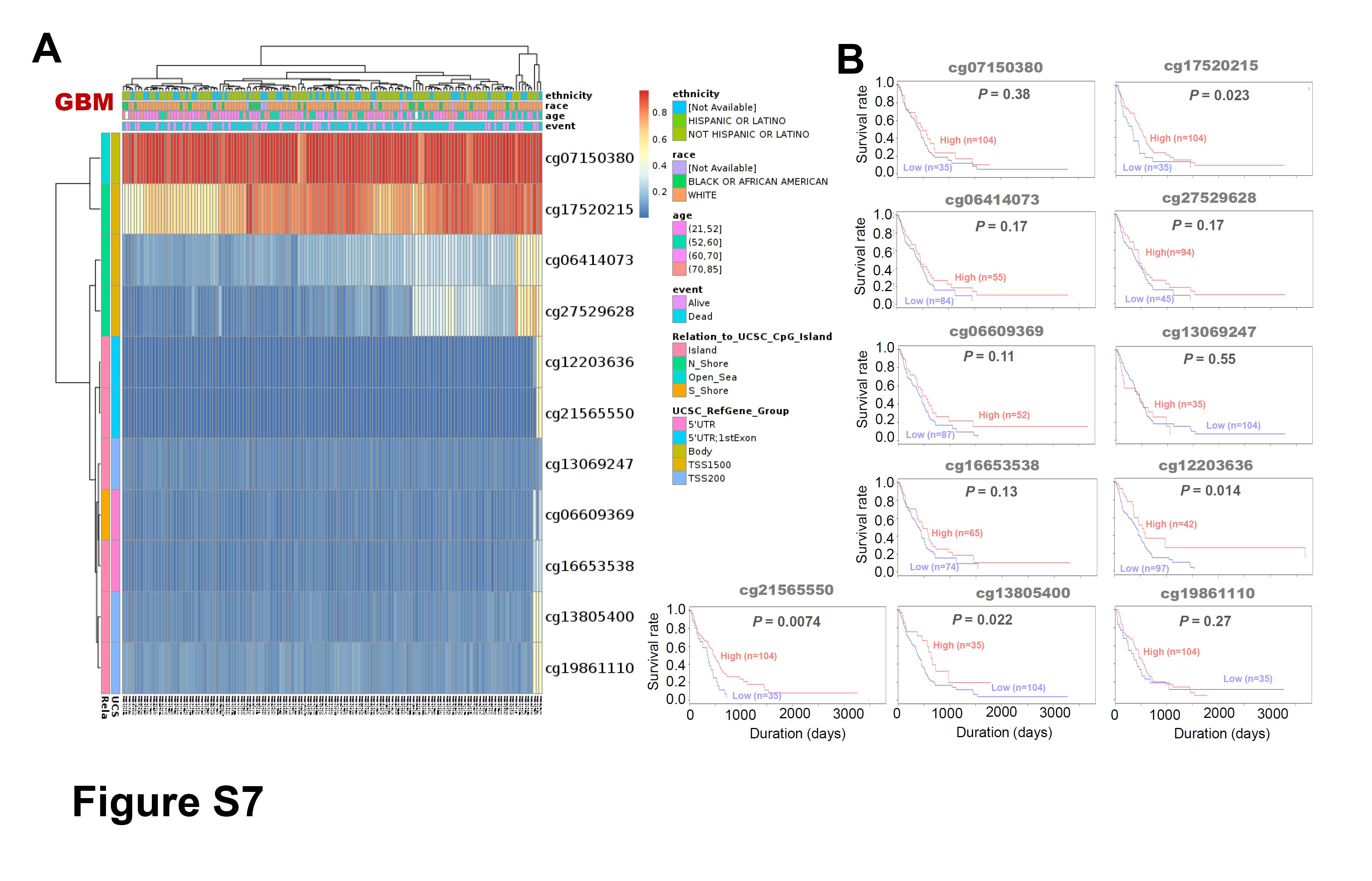

Supplement: Supplementary file 7 — Fig S7 [file CAM4-10-2826-s003.tif]

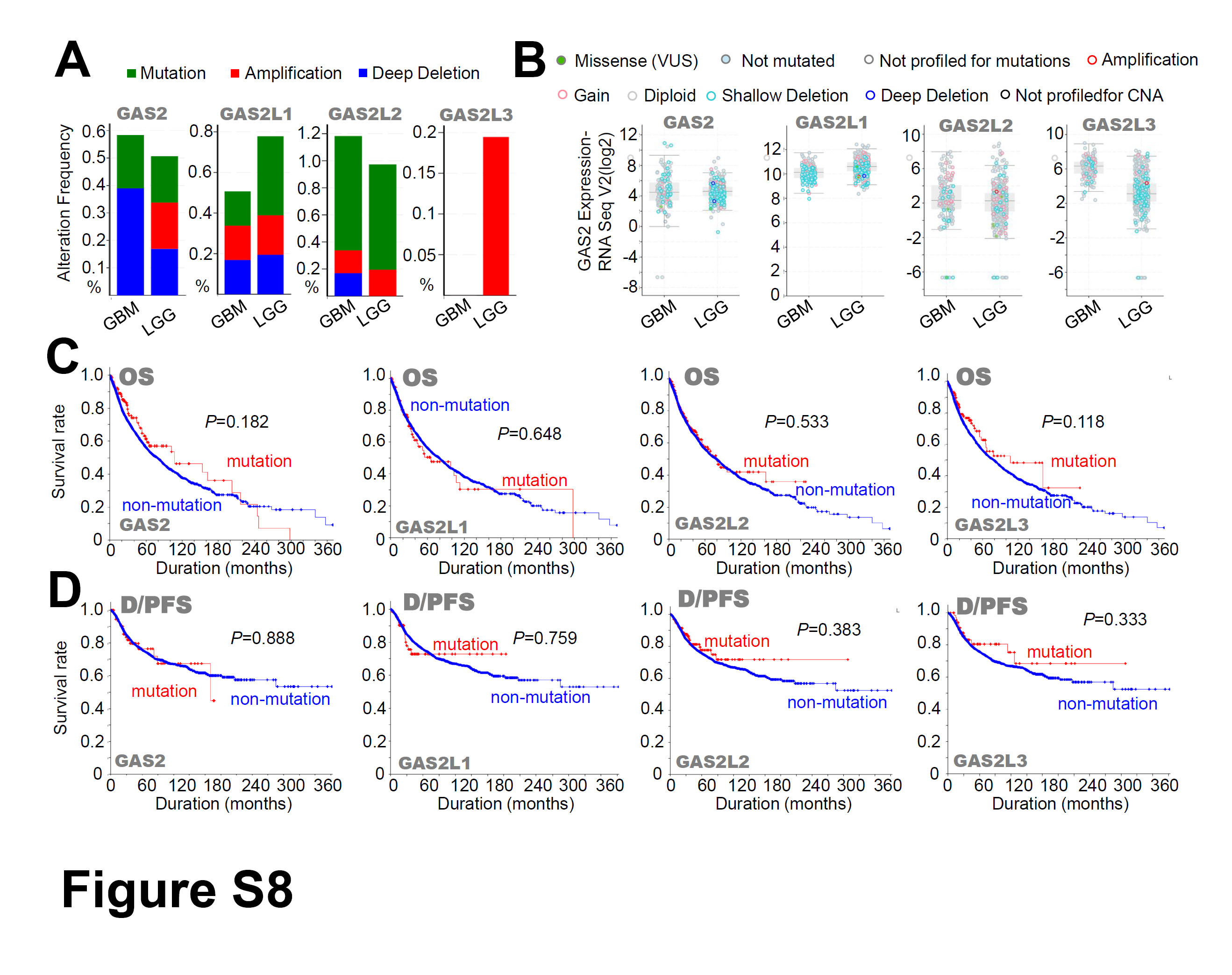

Supplement: Supplementary file 8 — Fig S8 [file CAM4-10-2826-s002.tif]

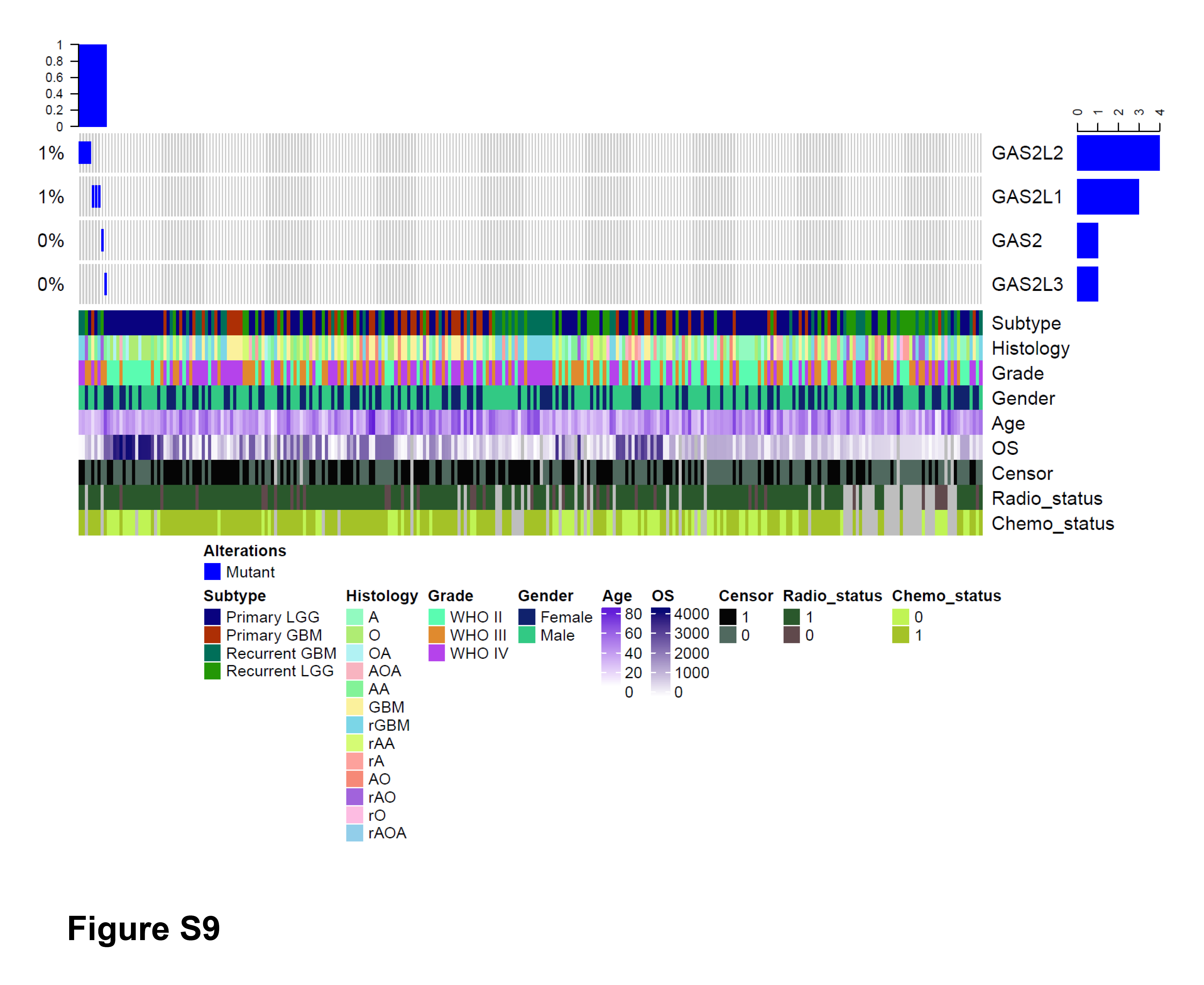

Supplement: Supplementary file 9 — Fig S9 [file CAM4-10-2826-s004.tif]

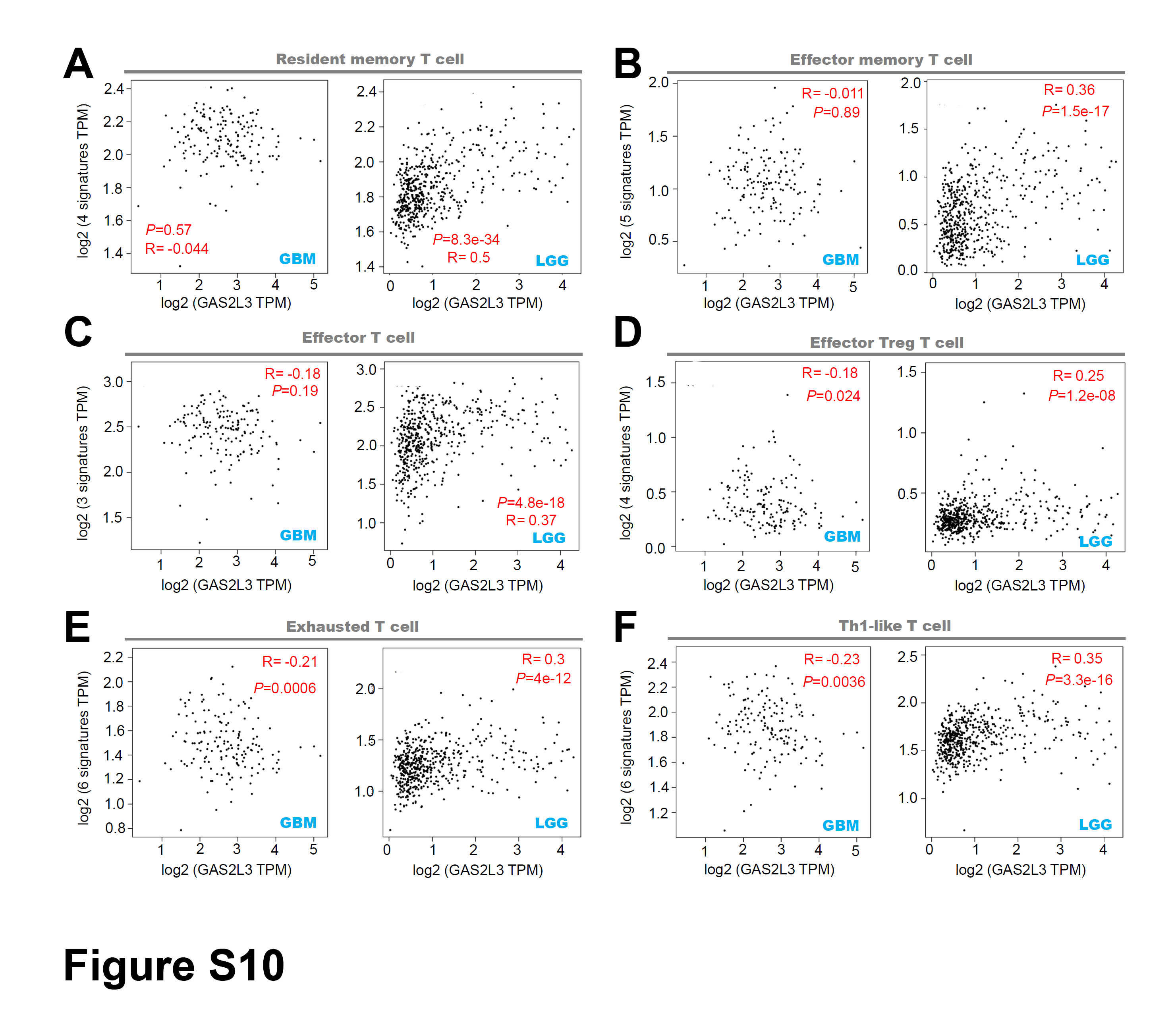

Supplement: Supplementary file 10 — Fig S10 [file CAM4-10-2826-s011.tif]

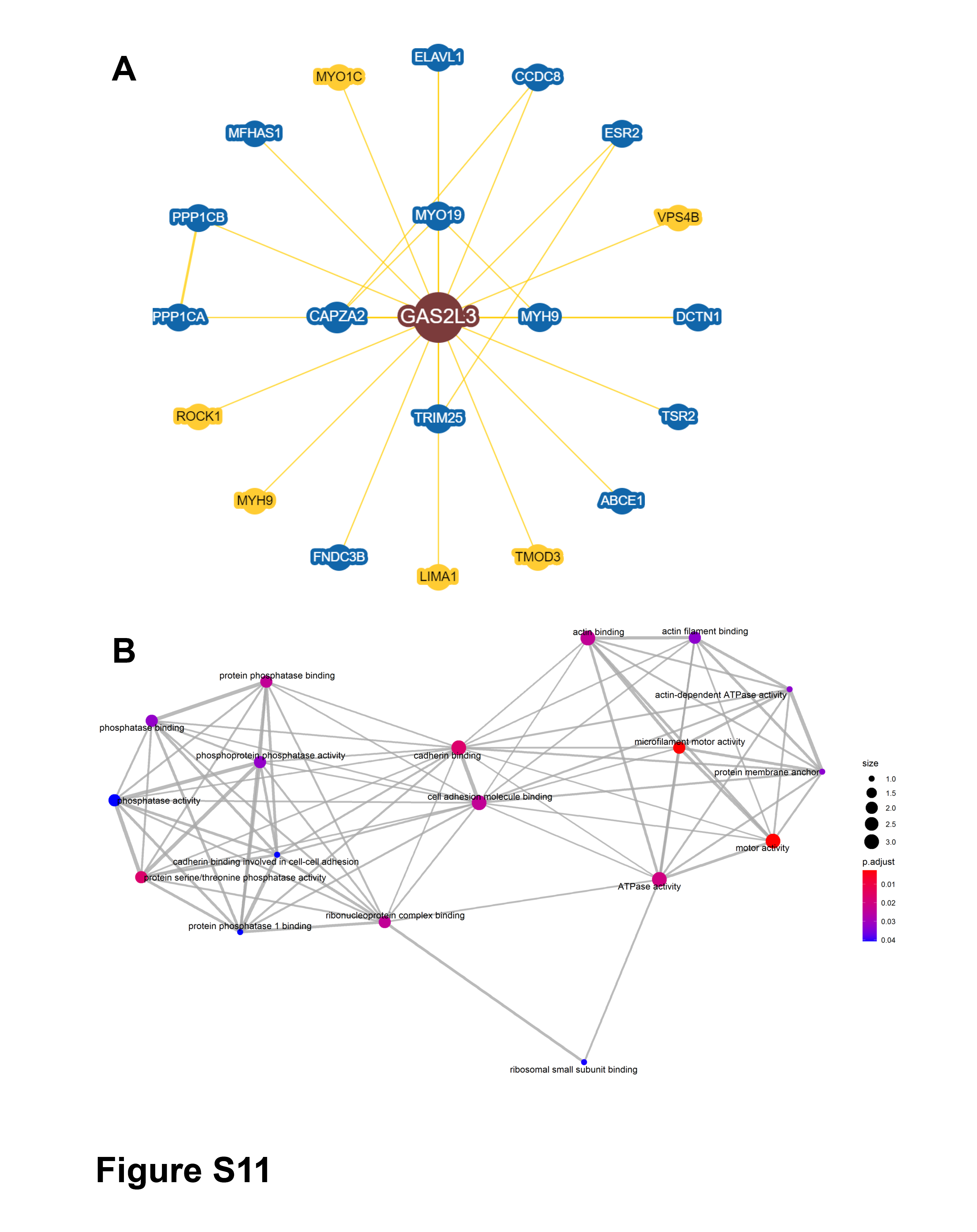

Supplement: Supplementary file 11 — Fig S11 [file CAM4-10-2826-s008.tif]
