# Supplementary material for: Integrative analysis regarding the correlation between GAS2 family genes and human glioma prognosis
Source: Cancer Med. 2021 Mar 12;10(8):2826–39. doi: 10.1002/cam4.3829 (PMC8026934; doi:10.1002/cam4.3829)
Supplement: Supplementary file 12 — Table S1 [file CAM4-10-2826-s010.doc]

## TABLE S1 Correlation of GAS2 family gene expression and clinical prognosis of glioma cases of TCGA（Cox proportional hazard model）

| **Gene** | **Disease** | **coef** | **HR** | **95% CI_up** | **95% CI_down** | ***Cox_P**** |
| --- | --- | --- | --- | --- | --- | --- |
| ***GAS2*** | LGG | 0.077 | 1.081 | 0.882 | 1.324 | 0.454 |
|  | GBM | 0.008 | 1.008 | 0.921 | 1.103 | 0.861 |
| ***GAS2L1*** | LGG | -0.444 | 0.642 | 0.457 | 0.901 | 0.010 |
|  | GBM | -0.005 | 0.995 | 0.850 | 1.165 | 0.949 |
| ***GAS2L2*** | LGG | 0.273 | 1.315 | 1.161 | 1.488 | <0.001 |
|  | GBM | -0.017 | 0.983 | 0.887 | 1.090 | 0.743 |
| ***GAS2L3*** | LGG | 0.423 | 1.526 | 1.358 | 1.715 | <0.001 |
|  | GBM | 0.117 | 1.124 | 0.932 | 1.356 | 0.223 |

HR, hazard ratio; CI, confidence interval; LGG, brain low-grade glioma; GBM, glioblastoma multiforme.

*The covariables of age, gender, race, and tumor purity were used in the Cox proportional hazard model
